# Supplementary material for: Molecular Classification and Overcoming Therapy Resistance for Acute Myeloid Leukemia with Adverse Genetic Factors
Source: Int J Mol Sci. 2022 May 25;23(11):5950. doi: 10.3390/ijms23115950 (PMC9180585; doi:10.3390/ijms23115950)
Supplement: Supplementary file 1 [file ijms-23-05950-s001.zip › ijms-1704421-supplementary.pdf]

Supplemental Table S1. Clinical and cytogenetic characteristics

	2017 ELN risk group classified with conventional cytogenetic tests plus NGS		
	Non-adverse	Adverse	Total
Patient number	n = 66	n = 102	n = 168
Age (years), median (range)	56 (20-91)	64 (25-89)	63 (20-91)
Age ≥ 60	30 (45.5%)	64 (62.7%)	97 (57.7%)
Male sex	44 (67%)	67 (66%)	111 (66%)
Unfit newly diagnosed AML	25 (37.9%)	41 (40.2%)	66 (39.3%)
Relapsed/refractory AML	41 (62.1%)	61 (59.8%)	102 (60.7%)
Disease type			
De novo AML	56 (84.8%)	61 (59.8%)	117 (69.6%)
AML with MRC	7 (10.6%)	34 (33.3%)	41 (24.4%)
Therapy-related AML	3 (4.6%)	7 (6.9%)	10 (6.0%)
Treatment after enrollment			
intensive chemotherapy	19 (28.8%)	22 (21.6%)	41 (24.4%)
non-intensive treatment	41 (62.1%)	68 (66.7%)	109 (64.9%)
best supportive care	6 (9.1%)	10 (9.8%)	16 (9.5%)
missing data	0 (0%)	2 (1.9%)	2 (1.2%)
2017 ELN risk group classified only with conventional cytogenetic tests			
Non-adverse	66 (100%)	39 (38.2%)	105 (62.5%)
Adverse	0 (0%)	63 (61.8%)	63 (37.5%)
Detected cytogenetic abnormality			

NPM1	38 (57.6%)	3 (2.9%)	41 (24.4%)
FLT3-ITD	14 (21.2%)	16 (15.7%)	30 (17.9%)
t(8;21)(q22;q22.1); *RUNX1-RUNX1T1*	11 (16.7%)	3 (2.9%)	14 (8.3%)
inv(16)(p13.1q22) or t(16;16)(p13.1;q22); *CBFB- MYH1*1	7 (10.6%)	0 (0%)	7 (4.2%)
t(9;11)(p21.3;q23.3); *MLLT3-KMT2A*	1 (1.5%)	1 (1.0%)	2 (1.2%)
Complex karyotype or monosomal karyotype or -5/del(5q) or -7 or -17/abn(17p)	0 (0%)	39 (38.2%)	39 (23.2%)
TP53	0 (0%)	36 (35.2%)	36 (21.4%)
ASXL1	0 (0%)	23 (22.5%)	23 (13.7%)
RUNX1	0 (0%)	21 (20.6%)	21 (12.5%)
t(v;11q23.3); *KMT2A* rearranged	0 (0%)	11 (10.8%)	11 (6.5%)
inv(3)(q21.3q26.2) or t(3;3)(q21.3;q26.2); *GATA2,MECOM(EVI1)*	0 (0%)	9 (8.8%)	9 (5.3%)
t(9;22)(q34.1;q11.2); *BCR-ABL1*	0 (0%)	1 (1.0%)	1 (0.6%)
t(6;9)(p23;q34.1); *DEK-NUP214*	0 (0%)	0 (0%)	0 (0%)
Outcome			
OS, median (months), (95% CI)	35.35 (24.01-NA)	17.05 (12.02-29.66)	24.80 (19.97-36.82)
PFS, median (months), (95% CI)	21.97 (17.05-36.30)	10.44 (7.65-19.74)	17.57 (11.26-22.47)
CR/CRi rate	39 (59.1%)	26 (25.5%)	65 (38.7%)
SCT after enrollment	22 (33.3%)	37 (36.3%)	59 (35.1%)

AML; acute myeloid leukemia, MRC; myelodysplasia-related changes, ELN; European LeukemiaNet, OS; overall survival, PFS; progression free survival, CR; complete response, CRi; complete remission with incomplete hematological recovery, SCT; stem cell transplant, CI; confidence interval, NGS; next-generation sequencing.
